# 4-[2-(2-Benzyl­idene­hydrazin­ylidene)-3,6-dihydro-2*H*-1,3,4-thia­diazin-5-yl]-3-(4-meth­oxy­phen­yl)­sydnone

**DOI:** 10.1107/S1600536811009329

**Published:** 2011-03-19

**Authors:** Hoong-Kun Fun, Madhukar Hemamalini, Nithinchandra, Balakrishna Kalluraya

**Affiliations:** a X-ray Crystallography Unit, School of Physics, Universiti Sains Malaysia, 11800 USM, Penang, Malaysia; b Department of Studies in Chemistry, Mangalore University, Mangalagangotri, Mangalore 574 199, India

## Abstract

In the title compound, C_19_H_16_N_6_O_3_S, the 3,6-dihydro-1,3,4-thia­diazine ring adopts a twist-boat conformation. The dihedral angle between the meth­oxy-substituted benzene ring and the oxadiazole ring is 71.91 (7)°. In the crystal structure, centrosymmetrically related mol­ecules are linked into dimers *via* pairs of inter­molecular N—H⋯N hydrogen bonds, generating *R*_2_^2^(8) ring motifs. There is an intra­molecular C—H⋯O hydrogen bond which generates an *S*(6) ring motif.

## Related literature

For applications of sydnones, see: Baker *et al.* (1949 ▶); Hedge *et al.* (2008 ▶); Rai *et al.* (2008 ▶); Kalluraya *et al.* (2003 ▶). For the definition of ring-puckering parameters, see: Cremer & Pople (1975 ▶). For the definition of graph-set notation, see: Bernstein *et al.* (1995 ▶). 
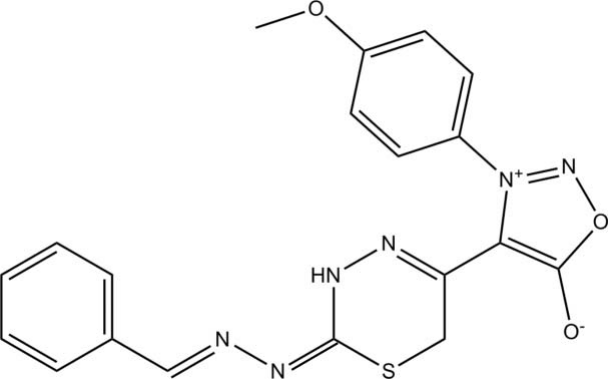


## Experimental

### 

#### Crystal data

C_19_H_16_N_6_O_3_S
*M* *_r_* = 408.44
Monoclinic, 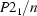

*a* = 14.9236 (15) Å
*b* = 5.9331 (7) Å
*c* = 21.425 (2) Å
β = 99.338 (2)°
*V* = 1871.9 (4) Å^3^
*Z* = 4
Mo *K*α radiation
μ = 0.21 mm^−1^
*T* = 296 K
0.77 × 0.07 × 0.04 mm

#### Data collection

Bruker APEXII DUO CCD area-detector diffractometer
Absorption correction: multi-scan (*SADABS*; Bruker, 2009 ▶) *T*_min_ = 0.856, *T*_max_ = 0.992
15668 measured reflections
4269 independent reflections
2889 reflections with *I* > 2σ(*I*)
*R*_int_ = 0.062

#### Refinement

*R*[*F* ^2^ > 2σ(*F* ^2^)] = 0.043
*wR*(*F* ^2^) = 0.106
*S* = 1.03
4269 reflections
267 parameters
H atoms treated by a mixture of independent and constrained refinement
Δρ_max_ = 0.37 e Å^−3^
Δρ_min_ = −0.29 e Å^−3^

### 

Data collection: *APEX2* (Bruker, 2009 ▶); cell refinement: *SAINT* (Bruker, 2009 ▶); data reduction: *SAINT*; program(s) used to solve structure: *SHELXTL* (Sheldrick, 2008 ▶); program(s) used to refine structure: *SHELXTL*; molecular graphics: *SHELXTL*; software used to prepare material for publication: *SHELXTL* and *PLATON* (Spek, 2009 ▶).

## Supplementary Material

Crystal structure: contains datablocks global, I. DOI: 10.1107/S1600536811009329/rz2567sup1.cif

Structure factors: contains datablocks I. DOI: 10.1107/S1600536811009329/rz2567Isup2.hkl

Additional supplementary materials:  crystallographic information; 3D view; checkCIF report

## Figures and Tables

**Table 1:** Hydrogen-bond geometry (Å, °)

*D*—H⋯*A*	*D*—H	H⋯*A*	*D*⋯*A*	*D*—H⋯*A*
N3—H1N3⋯N2^i^	0.87 (3)	2.04 (3)	2.905 (2)	173 (2)
C9—H9*B*⋯O2	0.97	2.39	3.057 (3)	126

Symmetry code: (i) 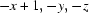.

**Fig. 1.**
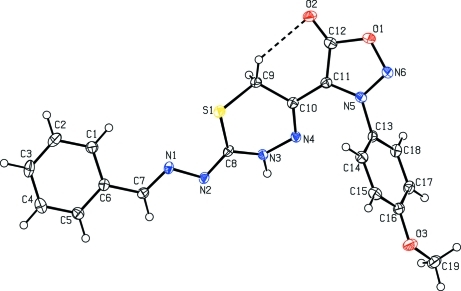
The asymmetric unit of the title compound, showing 30% probability displacement ellipsoids. The intramolecular hydrogen bond is shown as a dashed line.

**Fig. 2.**
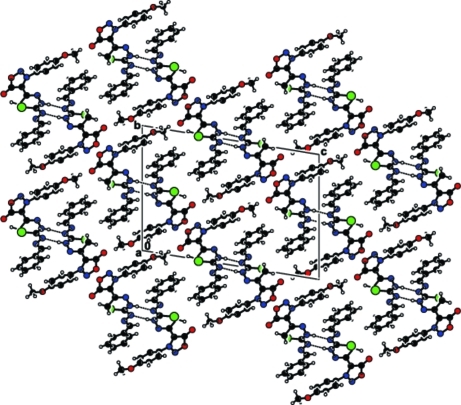
The crystal packing of the title compound viewed along the a axis. Intermolecular hydrogen bonds are shown as dashed lines.

## supplementary crystallographic information

### Comment

Sydnones constitute a well defined class of mesoionic compounds consisting of 1,2,3-oxadiazole ring system. The introduction of the concept of mesoionic structure for certain heterocyclic compounds in the year 1949 has proved to be a fruitful development in heterocyclic chemistry (Baker *et al.*, 1949). The study of sydnones still remains a field of interest because of their electronic structures and also because of the various types of biological activities displayed by some of them. Interest in sydnone derivatives has also been encouraged by the discovery that they exhibit various pharmacological activities (Hedge *et al.*, 2008; Rai *et al.*, 2008). Encouraged by these reports and in continuation of our research for biologically active nitrogen-containing heterocycles, a thiadiazine moiety at the 4-position of the phenylsydnone was introduced. A series of thiadiazines were synthesized by the condensation of 4-bromoacetyl-3-arylsydnones with N'-[phenylmethylidene]carbonohydrazide. 4-Bromoacetyl-3-arylsydnones were in turn obtained by the photochemical bromination of 4-acetyl-3-arylsydnones (Kalluraya *et al.*, 2003).

In the title compound (Fig. 1), the 3,6-dihydro-1,3,4-thiadiazine ring (C8–C10/N3/N4/S1) adopts twist-boat conformation, with puckering parameters Q = 0.5852 (18) Å, Θ = 109.67 (18)° and φ = 138.84 (19)° (Cremer & Pople, 1975). The dihedral angle between the methoxy-substituted benzene ring (C13–C18) and the oxadiazole ring (C11–C12/O1/N5–N6) is 71.91 (7)°. In the crystal structure (Fig. 2), centrosymmetrically related molecules are linked into dimers *via* pairs of intermolecular N—H···N hydrogen bonds, generating an *R*^2^_2_(8) ring motif. There is an intramolecular C—H···O hydrogen bond which generates an *S*(6) ring motif.

### Experimental

To a mixture of 4-bromoacetyl-3-(*p*-anisyl)sydnone (0.01 mol) and N'-(phenylmethylidene) carbonohydrazide (0.01 mol) in ethanol, a catalytic amount of anhydrous sodium acetate was added. The solution was stirred at room temperature for 2–3 hours. The solid product that separated out was filtered and dried. It was then recrystallized from ethanol. Crystals suitable for X-ray analysis were obtained by slow evaporation of a DMF/ethanol solution (1:2 *v*/*v*).

### Refinement

Atom H1N3 was located from a difference Fourier map and refined freely [N—H = 0.87 (2) Å]. The remaining H atoms were positioned geometrically [C—H = 0.93–0.97 Å] and were refined using a riding model, with *U*_iso_(H) = 1.2 *U*_eq_(C) or 1.5 *U*_eq_(C) for methyl H atoms.

### Crystal data

**Table:**

C_19_H_16_N_6_O_3_S	*F*(000) = 848
*M**_r_* = 408.44	*D*_x_ = 1.449 Mg m^−^^3^
Monoclinic, *P*2_1_/*n*	Mo *K*α radiation, λ = 0.71073 Å
Hall symbol: -P 2yn	Cell parameters from 1908 reflections
*a* = 14.9236 (15) Å	θ = 3.0–27.3°
*b* = 5.9331 (7) Å	µ = 0.21 mm^−^^1^
*c* = 21.425 (2) Å	*T* = 296 K
β = 99.338 (2)°	Needle, orange
*V* = 1871.9 (4) Å^3^	0.77 × 0.07 × 0.04 mm
*Z* = 4	

### Data collection

**Table:**

Bruker APEXII DUO CCD area-detector diffractometer	4269 independent reflections
Radiation source: fine-focus sealed tube	2889 reflections with *I* > 2σ(*I*)
graphite	*R*_int_ = 0.062
φ and ω scans	θ_max_ = 27.5°, θ_min_ = 1.6°
Absorption correction: multi-scan (*SADABS*; Bruker, 2009)	*h* = −19→19
*T*_min_ = 0.856, *T*_max_ = 0.992	*k* = −7→7
15668 measured reflections	*l* = −27→27

### Refinement

**Table:**

Refinement on *F*^2^	Primary atom site location: structure-invariant direct methods
Least-squares matrix: full	Secondary atom site location: difference Fourier map
*R*[*F*^2^ > 2σ(*F*^2^)] = 0.043	Hydrogen site location: inferred from neighbouring sites
*wR*(*F*^2^) = 0.106	H atoms treated by a mixture of independent and constrained refinement
*S* = 1.03	*w* = 1/[σ^2^(*F*_o_^2^) + (0.0401*P*)^2^ + 0.4767*P*] where *P* = (*F*_o_^2^ + 2*F*_c_^2^)/3
4269 reflections	(Δ/σ)_max_ = 0.001
267 parameters	Δρ_max_ = 0.37 e Å^−^^3^
0 restraints	Δρ_min_ = −0.29 e Å^−^^3^

### Special details

**Table:**

Geometry. All s.u.'s (except the s.u. in the dihedral angle between two l.s. planes) are estimated using the full covariance matrix. The cell s.u.'s are taken into account individually in the estimation of s.u.'s in distances, angles and torsion angles; correlations between s.u.'s in cell parameters are only used when they are defined by crystal symmetry. An approximate (isotropic) treatment of cell s.u.'s is used for estimating s.u.'s involving l.s. planes.
Refinement. Refinement of F^2^ against ALL reflections. The weighted R-factor wR and goodness of fit S are based on F^2^, conventional R-factors R are based on F, with F set to zero for negative F^2^. The threshold expression of F^2^ > 2σ(F^2^) is used only for calculating R-factors(gt) etc. and is not relevant to the choice of reflections for refinement. R-factors based on F^2^ are statistically about twice as large as those based on F, and R- factors based on ALL data will be even larger.

### Fractional atomic coordinates and isotropic or equivalent isotropic displacement parameters (Å^2^)

**Table:**

	*x*	*y*	*z*	*U*_iso_*/*U*_eq_	
S1	0.51196 (3)	0.06066 (11)	0.18109 (2)	0.02408 (16)	
O1	0.29051 (9)	−0.7158 (3)	0.24009 (6)	0.0274 (4)	
O2	0.43051 (9)	−0.5875 (3)	0.28329 (6)	0.0261 (4)	
O3	0.03886 (9)	−0.2449 (3)	−0.06332 (6)	0.0302 (4)	
N1	0.58722 (11)	0.3462 (3)	0.10107 (7)	0.0197 (4)	
N2	0.53281 (10)	0.1897 (3)	0.06401 (7)	0.0203 (4)	
N3	0.45763 (11)	−0.1430 (3)	0.06974 (7)	0.0189 (4)	
N4	0.39709 (10)	−0.2843 (3)	0.09276 (7)	0.0185 (4)	
N5	0.26615 (11)	−0.5345 (3)	0.15329 (7)	0.0202 (4)	
N6	0.22703 (11)	−0.6811 (4)	0.18620 (7)	0.0264 (5)	
C1	0.73234 (13)	0.6171 (4)	0.16329 (9)	0.0242 (5)	
H1A	0.7205	0.4846	0.1837	0.029*	
C2	0.79578 (14)	0.7681 (5)	0.19293 (10)	0.0320 (6)	
H2A	0.8264	0.7369	0.2334	0.038*	
C3	0.81414 (15)	0.9647 (5)	0.16313 (11)	0.0339 (6)	
H3A	0.8574	1.0645	0.1833	0.041*	
C4	0.76819 (14)	1.0137 (5)	0.10314 (10)	0.0287 (6)	
H4A	0.7796	1.1476	0.0833	0.034*	
C5	0.70513 (13)	0.8620 (4)	0.07295 (9)	0.0230 (5)	
H5A	0.6753	0.8936	0.0323	0.028*	
C6	0.68565 (13)	0.6638 (4)	0.10230 (8)	0.0199 (5)	
C7	0.62003 (13)	0.5032 (4)	0.06982 (8)	0.0204 (5)	
H7A	0.6018	0.5142	0.0263	0.025*	
C8	0.50177 (12)	0.0383 (4)	0.09904 (8)	0.0181 (5)	
C9	0.49414 (13)	−0.2359 (4)	0.19578 (8)	0.0221 (5)	
H9A	0.5470	−0.3213	0.1886	0.026*	
H9B	0.4866	−0.2566	0.2395	0.026*	
C10	0.41158 (12)	−0.3220 (4)	0.15311 (8)	0.0191 (5)	
C11	0.34998 (13)	−0.4661 (4)	0.18046 (8)	0.0189 (5)	
C12	0.36813 (13)	−0.5822 (4)	0.23935 (8)	0.0220 (5)	
C13	0.21053 (12)	−0.4579 (4)	0.09497 (8)	0.0199 (5)	
C14	0.17263 (14)	−0.2452 (4)	0.09297 (9)	0.0240 (5)	
H14A	0.1857	−0.1480	0.1273	0.029*	
C15	0.11479 (14)	−0.1796 (5)	0.03878 (9)	0.0266 (5)	
H15A	0.0881	−0.0376	0.0365	0.032*	
C16	0.09668 (13)	−0.3280 (5)	−0.01245 (8)	0.0227 (5)	
C17	0.13592 (13)	−0.5395 (5)	−0.00988 (9)	0.0247 (5)	
H17A	0.1242	−0.6363	−0.0444	0.030*	
C18	0.19321 (13)	−0.6064 (4)	0.04498 (9)	0.0234 (5)	
H18A	0.2194	−0.7490	0.0478	0.028*	
C19	0.01814 (14)	−0.3879 (5)	−0.11771 (9)	0.0340 (7)	
H19A	−0.0240	−0.3128	−0.1496	0.051*	
H19B	−0.0084	−0.5258	−0.1059	0.051*	
H19C	0.0729	−0.4210	−0.1340	0.051*	
H1N3	0.4553 (16)	−0.161 (5)	0.0291 (12)	0.041 (7)*	

### Atomic displacement parameters (Å^2^)

**Table:**

	*U*^11^	*U*^22^	*U*^33^	*U*^12^	*U*^13^	*U*^23^
S1	0.0287 (3)	0.0285 (4)	0.0149 (2)	−0.0068 (3)	0.00304 (18)	−0.0044 (2)
O1	0.0302 (8)	0.0318 (12)	0.0200 (6)	−0.0042 (8)	0.0033 (6)	0.0077 (7)
O2	0.0281 (7)	0.0319 (12)	0.0177 (6)	0.0013 (8)	0.0016 (5)	0.0023 (7)
O3	0.0242 (7)	0.0414 (13)	0.0238 (7)	0.0037 (8)	0.0003 (6)	0.0051 (7)
N1	0.0198 (8)	0.0190 (12)	0.0199 (7)	−0.0040 (9)	0.0022 (6)	−0.0060 (8)
N2	0.0207 (8)	0.0229 (13)	0.0170 (7)	−0.0048 (9)	0.0018 (6)	−0.0034 (8)
N3	0.0239 (8)	0.0198 (12)	0.0134 (7)	−0.0055 (9)	0.0043 (6)	−0.0014 (7)
N4	0.0185 (8)	0.0200 (12)	0.0176 (7)	−0.0032 (8)	0.0046 (6)	−0.0006 (7)
N5	0.0213 (8)	0.0217 (12)	0.0186 (7)	−0.0008 (9)	0.0059 (6)	0.0017 (8)
N6	0.0282 (9)	0.0287 (14)	0.0218 (8)	−0.0087 (10)	0.0021 (7)	0.0045 (8)
C1	0.0245 (10)	0.0246 (16)	0.0233 (9)	0.0005 (11)	0.0030 (8)	0.0008 (10)
C2	0.0273 (11)	0.0363 (19)	0.0287 (10)	−0.0002 (13)	−0.0061 (9)	−0.0048 (11)
C3	0.0263 (11)	0.0294 (18)	0.0441 (12)	−0.0085 (12)	0.0003 (10)	−0.0118 (12)
C4	0.0258 (10)	0.0220 (16)	0.0404 (11)	−0.0015 (11)	0.0113 (9)	−0.0006 (11)
C5	0.0225 (10)	0.0235 (15)	0.0237 (9)	0.0006 (11)	0.0063 (8)	0.0005 (10)
C6	0.0188 (9)	0.0213 (15)	0.0203 (9)	−0.0011 (10)	0.0052 (7)	−0.0033 (9)
C7	0.0216 (9)	0.0217 (15)	0.0175 (8)	0.0005 (10)	0.0021 (7)	−0.0023 (9)
C8	0.0156 (8)	0.0227 (14)	0.0156 (8)	0.0003 (10)	0.0011 (7)	−0.0040 (9)
C9	0.0215 (9)	0.0277 (16)	0.0166 (8)	−0.0040 (11)	0.0018 (7)	0.0024 (9)
C10	0.0187 (9)	0.0217 (15)	0.0173 (8)	0.0001 (10)	0.0042 (7)	−0.0026 (9)
C11	0.0191 (9)	0.0207 (14)	0.0172 (8)	0.0008 (10)	0.0039 (7)	−0.0004 (9)
C12	0.0259 (10)	0.0223 (15)	0.0196 (9)	0.0026 (11)	0.0093 (8)	−0.0001 (9)
C13	0.0165 (9)	0.0244 (15)	0.0187 (8)	−0.0056 (10)	0.0026 (7)	−0.0009 (9)
C14	0.0274 (10)	0.0234 (15)	0.0219 (9)	−0.0004 (12)	0.0059 (8)	−0.0014 (10)
C15	0.0297 (11)	0.0238 (16)	0.0271 (10)	0.0012 (12)	0.0069 (8)	0.0014 (10)
C16	0.0161 (9)	0.0309 (17)	0.0215 (9)	−0.0018 (11)	0.0047 (7)	0.0035 (10)
C17	0.0235 (10)	0.0282 (16)	0.0226 (9)	−0.0046 (12)	0.0041 (8)	−0.0049 (10)
C18	0.0211 (9)	0.0241 (16)	0.0251 (9)	−0.0014 (11)	0.0039 (8)	−0.0023 (10)
C19	0.0268 (11)	0.051 (2)	0.0227 (10)	−0.0061 (13)	−0.0011 (8)	0.0030 (11)

### Geometric parameters (Å, °)

**Table:**

S1—C8	1.7448 (17)	C4—C5	1.384 (3)
S1—C9	1.814 (3)	C4—H4A	0.9300
O1—N6	1.385 (2)	C5—C6	1.387 (3)
O1—C12	1.406 (3)	C5—H5A	0.9300
O2—C12	1.212 (2)	C6—C7	1.459 (3)
O3—C16	1.368 (2)	C7—H7A	0.9300
O3—C19	1.434 (3)	C9—C10	1.500 (3)
N1—C7	1.289 (3)	C9—H9A	0.9700
N1—N2	1.394 (2)	C9—H9B	0.9700
N2—C8	1.303 (3)	C10—C11	1.447 (3)
N3—C8	1.360 (3)	C11—C12	1.425 (3)
N3—N4	1.381 (2)	C13—C18	1.379 (3)
N3—H1N3	0.87 (2)	C13—C14	1.381 (3)
N4—C10	1.295 (2)	C14—C15	1.385 (3)
N5—N6	1.314 (2)	C14—H14A	0.9300
N5—C11	1.354 (2)	C15—C16	1.399 (3)
N5—C13	1.456 (2)	C15—H15A	0.9300
C1—C2	1.381 (3)	C16—C17	1.382 (3)
C1—C6	1.405 (3)	C17—C18	1.394 (3)
C1—H1A	0.9300	C17—H17A	0.9300
C2—C3	1.378 (4)	C18—H18A	0.9300
C2—H2A	0.9300	C19—H19A	0.9600
C3—C4	1.386 (3)	C19—H19B	0.9600
C3—H3A	0.9300	C19—H19C	0.9600
			
C8—S1—C9	96.30 (10)	C10—C9—H9A	109.5
N6—O1—C12	111.11 (15)	S1—C9—H9A	109.5
C16—O3—C19	117.3 (2)	C10—C9—H9B	109.5
C7—N1—N2	114.88 (15)	S1—C9—H9B	109.5
C8—N2—N1	111.09 (15)	H9A—C9—H9B	108.1
C8—N3—N4	127.66 (15)	N4—C10—C11	119.49 (18)
C8—N3—H1N3	119.9 (18)	N4—C10—C9	122.47 (18)
N4—N3—H1N3	111.0 (17)	C11—C10—C9	117.88 (16)
C10—N4—N3	116.66 (16)	N5—C11—C12	105.40 (18)
N6—N5—C11	115.31 (16)	N5—C11—C10	127.34 (17)
N6—N5—C13	115.21 (16)	C12—C11—C10	127.04 (17)
C11—N5—C13	129.33 (17)	O2—C12—O1	120.48 (19)
N5—N6—O1	104.03 (15)	O2—C12—C11	135.4 (2)
C2—C1—C6	119.9 (2)	O1—C12—C11	104.12 (16)
C2—C1—H1A	120.0	C18—C13—C14	122.39 (19)
C6—C1—H1A	120.0	C18—C13—N5	118.4 (2)
C3—C2—C1	120.6 (2)	C14—C13—N5	119.06 (18)
C3—C2—H2A	119.7	C13—C14—C15	118.6 (2)
C1—C2—H2A	119.7	C13—C14—H14A	120.7
C2—C3—C4	120.1 (2)	C15—C14—H14A	120.7
C2—C3—H3A	120.0	C14—C15—C16	119.7 (2)
C4—C3—H3A	120.0	C14—C15—H15A	120.1
C5—C4—C3	119.6 (2)	C16—C15—H15A	120.1
C5—C4—H4A	120.2	O3—C16—C17	124.6 (2)
C3—C4—H4A	120.2	O3—C16—C15	114.5 (2)
C4—C5—C6	121.07 (19)	C17—C16—C15	120.89 (19)
C4—C5—H5A	119.5	C16—C17—C18	119.3 (2)
C6—C5—H5A	119.5	C16—C17—H17A	120.3
C5—C6—C1	118.7 (2)	C18—C17—H17A	120.3
C5—C6—C7	120.74 (18)	C13—C18—C17	119.0 (2)
C1—C6—C7	120.5 (2)	C13—C18—H18A	120.5
N1—C7—C6	120.39 (17)	C17—C18—H18A	120.5
N1—C7—H7A	119.8	O3—C19—H19A	109.5
C6—C7—H7A	119.8	O3—C19—H19B	109.5
N2—C8—N3	118.03 (16)	H19A—C19—H19B	109.5
N2—C8—S1	123.16 (17)	O3—C19—H19C	109.5
N3—C8—S1	118.79 (15)	H19A—C19—H19C	109.5
C10—C9—S1	110.72 (15)	H19B—C19—H19C	109.5
			
C7—N1—N2—C8	−179.44 (19)	N6—N5—C11—C10	174.4 (2)
C8—N3—N4—C10	−33.8 (3)	C13—N5—C11—C10	−10.3 (4)
C11—N5—N6—O1	−0.5 (2)	N4—C10—C11—N5	−14.9 (4)
C13—N5—N6—O1	−176.51 (17)	C9—C10—C11—N5	169.6 (2)
C12—O1—N6—N5	1.3 (2)	N4—C10—C11—C12	158.9 (2)
C6—C1—C2—C3	−0.2 (3)	C9—C10—C11—C12	−16.6 (3)
C1—C2—C3—C4	0.7 (4)	N6—O1—C12—O2	178.8 (2)
C2—C3—C4—C5	−1.3 (3)	N6—O1—C12—C11	−1.6 (2)
C3—C4—C5—C6	1.4 (3)	N5—C11—C12—O2	−179.3 (3)
C4—C5—C6—C1	−1.0 (3)	C10—C11—C12—O2	5.8 (4)
C4—C5—C6—C7	−179.12 (19)	N5—C11—C12—O1	1.2 (2)
C2—C1—C6—C5	0.4 (3)	C10—C11—C12—O1	−173.7 (2)
C2—C1—C6—C7	178.52 (19)	N6—N5—C13—C18	−71.5 (2)
N2—N1—C7—C6	−173.51 (17)	C11—N5—C13—C18	113.2 (3)
C5—C6—C7—N1	−164.7 (2)	N6—N5—C13—C14	104.6 (2)
C1—C6—C7—N1	17.2 (3)	C11—N5—C13—C14	−70.7 (3)
N1—N2—C8—N3	−171.36 (17)	C18—C13—C14—C15	0.3 (3)
N1—N2—C8—S1	10.1 (2)	N5—C13—C14—C15	−175.62 (17)
N4—N3—C8—N2	−158.81 (19)	C13—C14—C15—C16	−0.5 (3)
N4—N3—C8—S1	19.8 (3)	C19—O3—C16—C17	−0.3 (3)
C9—S1—C8—N2	−160.46 (18)	C19—O3—C16—C15	179.43 (17)
C9—S1—C8—N3	21.05 (18)	C14—C15—C16—O3	−179.88 (18)
C8—S1—C9—C10	−49.20 (14)	C14—C15—C16—C17	−0.1 (3)
N3—N4—C10—C11	179.15 (19)	O3—C16—C17—C18	−179.35 (17)
N3—N4—C10—C9	−5.5 (3)	C15—C16—C17—C18	1.0 (3)
S1—C9—C10—N4	48.2 (3)	C14—C13—C18—C17	0.5 (3)
S1—C9—C10—C11	−136.34 (18)	N5—C13—C18—C17	176.44 (17)
N6—N5—C11—C12	−0.4 (3)	C16—C17—C18—C13	−1.1 (3)
C13—N5—C11—C12	174.9 (2)		

### Hydrogen-bond geometry (Å, °)

**Table:**

*D*—H···*A*	*D*—H	H···*A*	*D*···*A*	*D*—H···*A*
N3—H1N3···N2^i^	0.87 (3)	2.04 (3)	2.905 (2)	173 (2)
C9—H9B···O2	0.97	2.39	3.057 (3)	126

Symmetry codes: (i) −*x*+1, −*y*, −*z*.
